# Pulmonary Metastasis of Grade 1 Meningioma Mimicking a Carcinoid Tumor: A Case Report with FDG PET/CT, Ga-68 DOTATATE PET/CT, and Pathological Confirmation

**DOI:** 10.1055/s-0046-1822664

**Published:** 2026-05-05

**Authors:** Xavier Rabbath, Cheng-Han Shih, Marc-André Morin

**Affiliations:** 1 Department of Nuclear Medicine, Centre Hospitalier de l'Université de Montréal (CHUM), Montreal, Quebec, Canada

**Keywords:** meningioma, metastasis, grade I, [18F]-FDG PET/CT, DOTATATE-PET

## Abstract

Meningioma metastasis is a rare complication of a common condition. This case report describes the first known instance of detection on both Fluorodeoxyglucose Positron Emission Tomography (FDG-PET) and DOTA-(Tyr ^3^ )-octreotate Positron Emission Tomography (DOTATATE-PET) of a pulmonary metastasis from grade I meningioma. A 68-year-old woman with grade I meningioma showed a pulmonary lesion post-surgery, with FDG-PET showing mild hypermetabolism and DOTATATE-PET revealing intense uptake, consistent with a well-differentiated carcinoid tumor. However, histopathological analysis confirmed grade I meningioma metastasis. This case highlights DOTATATE-PET's potential in screening for metastases, even in grade I meningiomas.

## Introduction

Although meningioma is a common tumor, metastatic dissemination is rare, especially for grade I tumors. 1 2 To our knowledge, this is among the first reported cases of pulmonary metastasis from a grade I meningioma to be detected by both Fluorodeoxyglucose Positron Emission Tomography (FDG-PET) and DOTA-(Tyr ^3^ )-octreotate Positron Emission Tomography (DOTATATE-PET) and confirmed by pathology. This case aims to highlight DOTATATE-PET's potential in screening for metastases, even in grade I meningiomas.

## Case Report

**Fig. 1:**
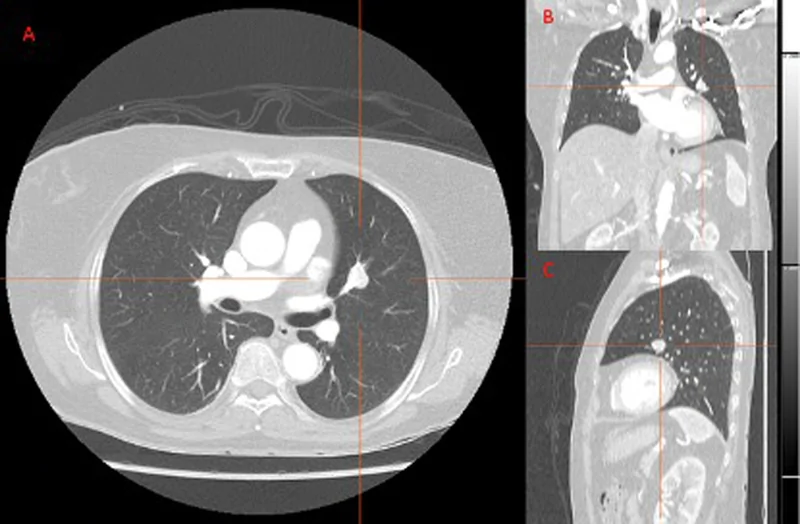
CT angiography on postoperative day 4 ( **A** ) axial CT, ( **B** ) coronal CT, ( **C** ) sagittal CT.

A 68-year-old woman with no significant medical history underwent subtotal resection of a falcine meningioma. Pathological analysis confirmed a WHO Grade I (benign) meningioma with no histologic or immunohistochemical markers of aggressiveness. However, FISH analysis revealed a 1p deletion, a finding associated with increased clinical aggressiveness. 3 A chest radiograph performed on postoperative day 2 revealed a dilated ascending aorta, which was further characterized by thoracic CT angiography on postoperative day 4 and incidentally revealed a slightly lobulated left upper lobe lesion measuring 17 × 13 mm ( Fig. 1 ).

**Fig. 2:**
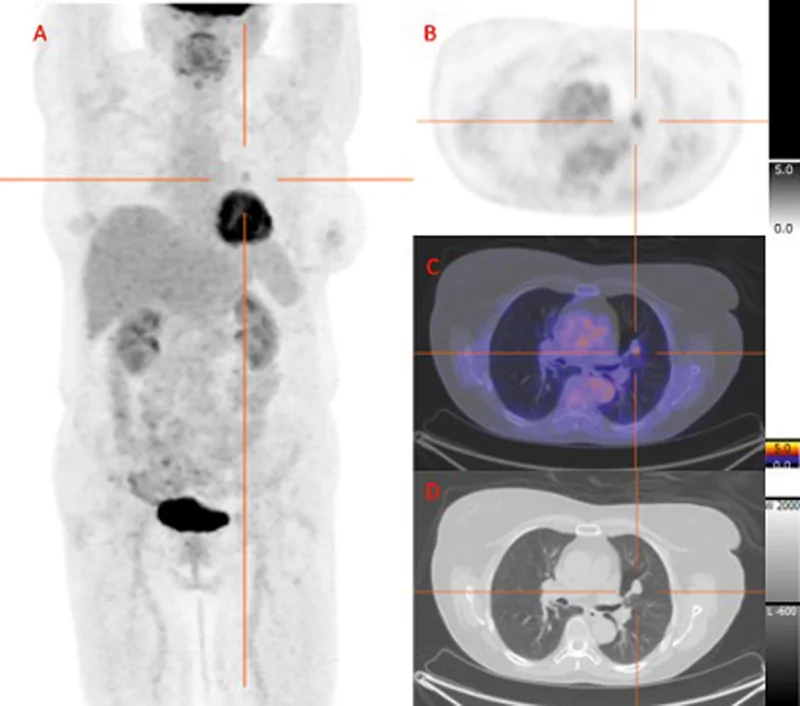
[18F]-FDG PET/CT 3 months later ( **A** ) maximum intensity projection PET, ( **B** ) axial PET, ( **C** ) fused axial PET/CT, ( **D** ) low-dose CT. [18F]-FDG PET/CT, Fluorine-18 Fluorodeoxyglucose Positron Emission Tomography/Computed Tomography.

[18F]-FDG PET/CT performed 3 months later revealed mild hypermetabolism of the pulmonary nodule (maximum standardized uptake value [SUVmax] 2.2), without associated lymphadenopathy or additional hypermetabolic foci ( Fig. 2 ).

A neoplastic lesion was suspected, and differential diagnoses included a carcinoid tumor considering low FDG uptake. Histopathological correlation or a DOTATATE-PET scan was suggested. Bronchoalveolar lavage, endobronchial brushing, and endobronchial ultrasound -guided lymph node biopsy did not reveal any neoplastic cells.

**Fig. 3:**
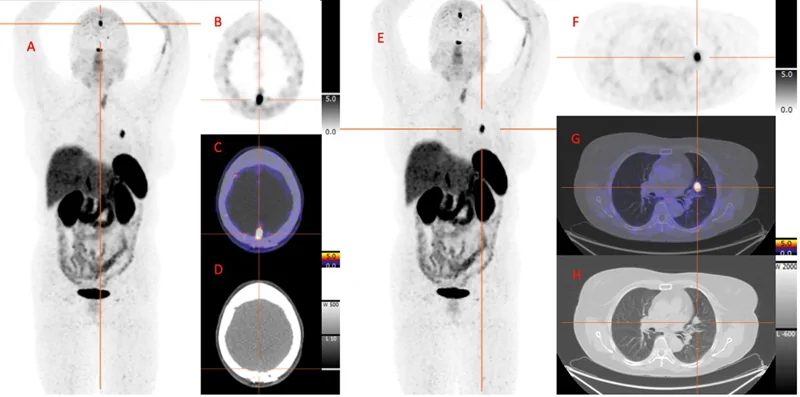
[68Ga]-DOTATATE-PET/CT ( **A** and **E** ) Maximum intensity projection PET, ( **B** and **F** ) axial PET, ( **C** and **G** ) fused axial PET-CT, ( **D** and **H** ) low-dose CT. [68Ga]-DOTATATE-PET/CT, Gallium-68 DOTA-(Tyr ^3^ )-octreotate Positron Emission Tomography/Computed Tomography.

[68Ga]-DOTATATE PET/CT demonstrated a focal area (8 × 11 mm) of high uptake in the posterior sagittal sinus, corresponding to known residual meningioma ( Fig. 3A–D ). The pulmonary lesion demonstrated intense somatostatin receptor expression (SUVmax 10.7, Krenning score 3) and was interpreted as consistent with a well-differentiated carcinoid tumor. No lymph node or distant metastases were identified ( Fig. 3E–H ).

The patient underwent left upper lobectomy within 2 weeks. Histopathological examination revealed a grade I meningioma morphologically consistent with the intracranial lesion, with a *K _i_* -67 index of 5 to 10%. This ruled out the working diagnosis of carcinoid tumor and confirmed a pulmonary metastasis from a WHO Grade I meningioma. The pulmonary lesion thus demonstrated mild FDG uptake and high DOTATATE avidity, paralleling the molecular profile of the primary intracranial tumor.

Follow-up chest CT performed 20 months after lobectomy showed no evidence of pulmonary recurrence but revealed sclerotic bone lesions, which have not yet been further investigated and could potentially be evaluated with PET imaging.

## Discussion

Extracranial metastases from meningiomas are exceedingly rare, with less than 1% of cases reported to metastasize. 1 Furthermore, only 33.9% of meningioma metastases are grade I. 2 Metastases from grade I meningioma have previously been reported in bone (10%), lung (40%), liver (10%), pleura (8%), cervical lymph nodes (10%), and the intraspinal region (12%). 2

Reports of meningioma lung metastasis detected by FDG-PET, as well as pulmonary, bone, and liver metastases detected by [68Ga]-DOTATATE and [68Ga]-DOTATOC PET, have been published in grade II (atypical) and grade III (anaplastic) meningiomas. 4 5 6 7 Distant metastases are considerably rarer in grade I (benign) meningiomas than in higher-grade tumors. To our knowledge, this is among the first reported cases of pulmonary metastasis from a grade I meningioma detected by both FDG-PET and DOTATATE-PET and confirmed histologically.

## Conclusion

This case highlights the value of molecular imaging and the potential for even histologically benign meningiomas to exhibit unexpected aggressive behavior. DOTATATE PET could provide value in screening for such metastases, as 31% of cases are clinically silent. 2
